# Molecular Complexity of Diffuse Large B-Cell Lymphoma: Can It Be a Roadmap for Precision Medicine?

**DOI:** 10.3390/cancers12010185

**Published:** 2020-01-11

**Authors:** Nicoletta Coccaro, Luisa Anelli, Antonella Zagaria, Tommasina Perrone, Giorgina Specchia, Francesco Albano

**Affiliations:** Department of Emergency and Organ Transplantation (D.E.T.O.), Hematology Section, University of Bari, 70124 Bari, Italy; nicoletta.coccaro@uniba.it (N.C.); luisa.anelli@uniba.it (L.A.); tommasinaperrone@virgilio.it (T.P.);

**Keywords:** diffuse large B-cell lymphoma (DLBCL), COO classification, next-generation sequencing (NGS), biomarker, liquid biopsy, targeted therapy

## Abstract

Diffuse large B-cell lymphoma (DLBCL) is the most common non-Hodgkin lymphoma; it features extreme molecular heterogeneity regardless of the classical cell-of-origin (COO) classification. Despite this, the standard therapeutic approach is still immunochemotherapy (rituximab plus cyclophosphamide, doxorubicin, vincristine, and prednisone—R-CHOP), which allows a 60% overall survival (OS) rate, but up to 40% of patients experience relapse or refractory (R/R) disease. With the purpose of searching for new clinical parameters and biomarkers helping to make a better DLBCL patient characterization and stratification, in the last years a series of large discovery genomic and transcriptomic studies has been conducted, generating a wealth of information that needs to be put in order. We reviewed these researches, trying ultimately to understand if there are bases offering a roadmap toward personalized and precision medicine also for DLBCL.

## 1. Introduction

Diffuse large B-cell lymphoma (DLBCL) comprises about 40% of all lymphomas, constituting the most prevalent type of non-Hodgkin lymphoma. It can arise de novo or result from the malignant transformation of a more indolent lymphoma. According to *The 2016 revision of the World Health Organization (WHO) classification of lymphoid neoplasms* there are 13 subtypes of lymphoma defined as specific entities, designating the rest as DLBCL not otherwise specified (NOS), which account for the vast majority of DLBCLs [1]. The standard treatment approach consists of immunochemotherapy (rituximab plus cyclophosphamide, doxorubicin, vincristine, and prednisone—R-CHOP), which guarantees an overall survival (OS) of more than 60% for DLBCL-NOS cases. In particular, a subgroup of young patients with favourable-prognosis disease can even achieve the same clinical benefit with fewer cycles of R-CHOP [2]. However, up to 40% of patients suffer relapse or refractory (R/R) disease [3] and for them the standard salvage approach consists of autologous stem cell transplantation, even if long-term disease control is achieved in fewer than 50% of cases [4]. Survival is particularly poor for patients relapsing within one year after R-CHOP with fewer than 15% of patients achieving a durable remission [5,6,7]. Recently chimeric antigen receptor (CAR) T-cell therapies have been approved as alternative curative options for patients with relapsing or refractory disease. CAR T cells represent a new class of cellular immunotherapy involving ex vivo genetic modification of patients’ T cells, triggering T-cell activation and cytotoxicity [8], that demonstrated good efficacy in B-cell malignancies treatment, including DLBCL [9,10]. In this context, a prevision of poor OS is attributed to relapsing cases and to patients with refractory disease [6] for which even CAR T-cell therapy fails [11].

Therefore, it is essential to search for clinical parameters and biomarkers that could help to better DLBCL patients’ characterization and stratification. Nowadays, thanks to the availability of comprehensive genomic and transcriptomic analyses a wealth of information is generated, rendering the concept of personalized therapy more realistic. In the attempt to put some order in the most recent discoveries on DLBCL research, we reviewed the latest experimental studies in this field, focusing on the most important findings helping in the management of lymphoma patients from the perspective of personalized medicine.

### 1.1. Standard Prognosticators for DLBCL

One of the most commonly used prognostic tools is the *International Prognostic Index* (IPI) [12], whose validity and reliability has been enhanced by several upgrades [13]. However, it evaluates only five clinical parameters (age, lactate dehydrogenase, performance status, number of extranodal sites, and Ann Arbor stage), without considering the biologic characteristics of the tumour.

The first and nowadays most commonly used biologic prognosticator of DLBCL tumours is the cell-of-origin (COO) determination based on gene expression profiling (GEP), which subdivides most DLBCL-NOS patients into two main categories, namely germinal center B-cell-like (GCB), if presenting with expression features similar to germinal center cells, and activated B-cell-like (ABC) DLBCL [14], when presenting features similar to activated B-lymphocytes. This subdivision is relevant for therapy and prognosis, as ABC cases show a worse outcome as regards progression-free survival (PFS) and OS after treatment with R-CHOP standard therapy [14,15,16] in comparison to GCB patients.

However, GEP through microarrays poses a challenge because it is available only for a small fraction of patients whose mRNA can be extracted from fresh or frozen tissues. The attempts to substitute GEP with immunohistochemistry (IHC) applicable to formalin-fixed, paraffin-embedded (FFPE) tissue samples [17,18,19,20,21,22] evidenced another series of inherent difficulties linked to the extreme variability of results, even when the same algorithm (Hans, Choi, Colomo, Muris, Pileri, or Tally) was applied [23]. Indeed, when the two techniques were compared, it was evident that the classification of DLBCL based on the COO was different. 

Five years ago a new approach named Lymph2Cx was proposed for GCB/ABC COO classification; based on a panel of 20 genes and applicable to mRNA extracted from FFPE tissue samples, it is conducted on the NanoString platform and replicates the results of conventional GEP, demonstrating its superiority to IHC algorithms in various series of DLBCL cases [24,25,26,27].

In the context of GEP, a German research group recently analysed data generated from expression microarray analyses of 873 different types of lymphoma, including DLBCL, with the purpose of clarifying their phenotypic characteristics [28]. Through this approach, the investigators demonstrated that the transcriptome panorama of B-cell lymphomas consists more in a continuum of expression states than of clearly separated phenotypes, and in which every layer represents a different lymphoma and individual cases [28]. Focusing on DLBCL, a series of models employed GEP for classifying patients: Monti et al. subdivided cases into three groups that were identified in oxidative phosphorylation, B-cell receptor/proliferation, and host response [29]; Dybkaer et al. isolated B cells from reactive tonsils and identified B-cell-associated gene signatures (BAGS), highlighting for each an association with molecular findings [30]. Other researchers instead focused their efforts applying GEP in the study of the microenvironment of lymphoma cells [31,32] and in the search of specific immune signatures [33]. Indeed, over time a series of NanoString-based expression assays has been suggested; for example, the Michaelsen group recently proposed a new NanoString-based assay for BAGS classification to overcome the difficulty of microarray-based GEP, in order to better categorize DLBCL in diverse B-cell subtypes [34]. Analysing microarray data from 970 patients belonging to four different cohorts, they first selected genes and then created and tested a new NanoString-based BAGS2Clinic assay for quick and easy-to-use BAGS profiling. Then, they tested the assay in an independent cohort of 88 lymph node biopsies and confirmed that it showed a good correspondence with the original BAGS classifier, having an overall accuracy of 84% and a subtype-specific accuracy ranging between 80% and 99%. It seems that BAGS classification could highlight important features of tumour biology and aspects about resistance to immuno- and chemotherapy that can be employed when choosing novel treatment strategies for DLBCL patients [34].

However, albeit meticulous, even COO classification through GEP reserves diverse exceptions, and variation in patient outcome persists even within each COO subtype, posing the main difficulty in management of patients and raising the question of the deep study of the extreme molecular heterogeneity that features DLBCL [35]. Consequently, several studies in the last years attempted to discover further stratification parameters serving to make a better patient categorization, and additional to the existing ones that presented some critical issues. Many prognostic markers have been considered, such as MYC gene alterations, which characterize from 5% to 15% of de novo DLBCL and confer a worse prognosis and higher risk of central nervous system involvement [36], as well as TP53 mutation, Epstein–Barr virus infection, CD5 expression, CD30 expression, BCL2 rearrangement or expression, MHC class II expression, and others [37,38,39,40,41,42,43,44,45,46,47,48], each presenting with contradicting data regarding their prognostic relevance. All things considered, at the current moment, genomic and transcriptomic profiling through next-generation sequencing (NGS) is surely the most powerful tool to investigate the molecular heterogeneity, as well as to find new potential biomarkers useful for diagnosis, risk determination, and treatment choice in DLBCL. However, in clinical practice, most investigations are conducted through the so-called “low-throughput techniques” (e.g., fluorescence in situ hybridization—FISH) and microarrays. Such approaches are acceptable if considering the old classification procedures that restricted the analyses to the search for a limited number of alterations, but now that fine molecular profiling of each individual case is possible, the implementation of NGS is becoming necessary also in the daily clinical workflow, maybe restricting the analyses to the search for specific molecular alterations.

### 1.2. Discovering New Prognostic Biomarkers and Models

In the last years a series of discovery NGS researches was conducted trying to collect sequencing-derived information and to find focal alterations predicting prognosis, or in some cases, to elaborate models for DLBCL classification and prognostication (Table 1). 

In a large comprehensive exome and transcriptome sequencing of 1001 DLBCL cases [49] Reddy and colleagues identified a set of 150 driver genes, most of which were then functionally characterized with an unbiased CRISPR screen of DLBCL cell lines to define oncogenes promoting cell growth. Through this information they drew up a prognostic model based on the presence of genetic alterations that was found to be better than current prognostic methods such as COO determination, IPI, and dual MYC and BCL2 expression. According to this model, genetic and/or expression aberrations of MYC defined the patient group with the worst prognosis; on the contrary, CD70 alterations in GCB-DLBCLs characterized the group with the better outcome [49].

In a similar work by Schmitz and colleagues, NGS was adopted to unveil driver genes with recurrent alterations [50]. They performed whole-exome sequencing (WES), RNA-seq, gene copy number analysis and targeted resequencing of 372 genes of 574 DLBCL cases, mostly pre-treated (96.5%), of which 51.4% were ABC and 20% unclassified (non-ABC, non-GCB). They developed a specific algorithm to identify genetic subtypes on the basis of the co-occurrence of genetic aberrations, and defined four prevalent subtypes that they termed MCD (co-occurrence of MYD88L265P and CD79B mutations), BN2 (BCL6 fusions and NOTCH2 mutations), N1 (NOTCH1 mutations), and EZB (EZH2 mutations and BCL2 translocations). Each subtype featured differences in gene-expression signatures, sensitivity to immunochemotherapy and outcome, BN2 and EZB subtypes being associated with favourable survival, and MCD and N1 subtypes to inferior outcomes. Indeed, MCD and BN2 subtypes seemed to depend on a chronic active BCR signalling pathway, opening an option for targeted therapeutic inhibition [50].

Another similar 2018 research by Chapuy et al. analysed 304 samples from DLBCL patients to find recurrent mutations, low-frequency alterations, somatic copy number alterations, and structural variants [51] identifying a series of genetic drivers that led to another new molecular classification. Through consensus clustering they characterized five DLBCL subsets, which included a novel group of ABC-DLBCL lymphomas of extrafollicular/marginal zone origin with low-risk and associated to NOTCH2 mutations (C1); a group of ABC-DLBCL with gains in BCL2 and/or mutations in MYD88L265P, CD79B, PIM1, and PRDM1, and associated with an unfavourable outcome (C5); two distinct subsets of GCB-DLBCL with different outcomes and targetable alterations (C3 group with aberrations affecting PTEN and epigenetic mediators such as KMT2D, CREBBP, and EZH2 and poor outcomes; and C4 group with alterations of signal transducers such as BCR–PI3K, NF-κB, or RAS–JAK, of transcription activators such as BRAF and STAT3, in histone genes, and genes involved in immune evasion (CD83, CD70, and CD58), and with favourable outcomes); and an ABC/GCB-independent group with biallelic inactivation of TP53, CDKN2A loss, and genomic instability, associated with poor outcomes (C2). The characteristics of the five subgroups identified also correlate with outcome in an independent manner in regard to IPI, suggesting new chances of therapeutic options and providing a roadmap for the identification of actionable DLBCLs [51].

Comparing the last two proposed molecular classifications it seems that the C1, C3, and C5 groups overlap with the BN2, EZB, and MCD groups of the work by Schmitz et al [50]. However, there are also differences, such as the C2 and C4 groups that did not present similarities with those in the other research. Indeed, both classifications differed from the molecular classification by Reddy and colleagues, in which MYC status was correlated with clinical outcome [49]. Indeed, each model presents some critical issues: the model by Reddy et al. gives a prognostic value to each individual marker assessed, but, given the lack of clear clusters, it is hard to use for therapeutic purposes. The drawback of the study by Schmitz is that is only focuses on ABC type; the study of Chapuy et al. instead encompasses all DLBCL, however conformational studies are still lacking.

The utility of investigating only MYD88/CD79B mutations to improve DLBCL classification and prognostication was explored in a set of 250 DLBCL cases [52]. The authors analyzed MYD88/CD79B mutations through NGS or allele-specific PCR, MYC/BCL2/BCL6 rearrangements by FISH, and EBV infections by EBER-ISH, identifying MYD88 and CD79B mutations in 29.6% and 12.3%, MYC, BCL2, and BCL6 rearrangements in 10.6%, 13.6%, and 20.3%, and EBV in 11.7% of cases, respectively. This study revealed that MYD88-mutated cases presented a significantly inferior five-year OS compared to wild-type; indeed, patients without any of the analysed alterations showed a superior OS compared to others carrying at least one aberrancy. In multivariable analysis, evaluating clinical-pathologic characteristics, outcome, and prognosis according to IPI, MYD88 mutations retained the adverse prognostic impact. Thus, investigating MYD88 mutations in DLBCL presents clinical utility as they feature a distinct molecular subtype with adverse prognosis [52].

Further, MYD88 mutations, together with CD79A, CD79B, and CARD11 aberrations, are known to trigger chronic activation of the B-cell receptor (BCR) signalling pathway [65,66,67,68]. Immunoglobulin M (IgM) is expressed in 90% of the ABC-like DLBCL subtype, and together with CD79A and CD79B, constitutes the BCR signalling complex, another mechanism of BCR aberrant chronic activation [69]. A recent DNA copy number analysis of 1000 DLBCL cases identified gains of 18q21.2 as the most frequent genetic alteration in the ABC-like group, and recognized the TCF4 (E2-2) transcription factor gene as the main target of the genomic aberrations [53]. With in vitro and in vivo experiments the effects of TCF4 overexpression were studied, observing its binding to IGHM and MYC gene enhancers and the augmented expression of the corresponding transcripts and proteins. Indeed, it was demonstrated that inhibition of TCF4 activity through the BET inhibitor ARV771 triggered death of the ABC-like DLBCL cells. Thus, this information represents a rationale for the employment of BET inhibitors for the subset of patients carrying this alteration [53].

All the above-mentioned studies allowed the use of genomic aberrations to identify subgroups associated with distinct clinical outcomes, but it is not always possible offer a genetic classification to all DLBCL patients. In the attempt to solve this problem a series of other researches have been carried out. A 2019 study by Wang et al. employed WES data to establish mutant-allele tumour heterogeneity (MATH) [57]. Based on the median expression level, patients were divided into low and high MATH score classes in which the higher MATH score group was associated with a higher risk of progression as compared to a lower MATH score, both in the discovery and in the validation set. The authors conclude that MATH has a prognostic value that could be considered in the management of DLBCL patients; a higher score of MATH has proven to be an independent risk prognostic factor in predicting recurrence [57].

Another recent work instead studied the somatic hypermutation (SHM) mutational activities showing that they delineated the COO in DLBCL [55]. Normally SHM acts during B-cell development targeting an immunoglobulin variable region [70]; altered SHM hits several of the DLBCL driver genes [70,71,72]. Alkodsi et al. found that the expression of 36 SHM target genes featured four novel SHM subtypes, strongly associated and overlapping with genetic subtypes already characterized by Schmitz et al., and that were significantly associated with OS and PFS of DLBCL patients treated with immunochemotherapy [55]. Their stratification separates the GCB-DLBCL class into two major subtypes: SHM1, characterized by a high frequency of not always concurrent BCL2 and MYC aberrations and mutations in chromatin modifying genes, and including cases with poor outcome after standard R-CHOP therapy, that could be directed to alternative therapies; and SHM3, featuring mutations in the JAK-STAT pathway and a better outcome to standard cure. The ABC class was divided into SHM4, presenting with BCL6 fusions and mutations in CD70 and BCL10; and SHM2, presenting with the worst outcome and characterized by mutations in the BCR signalling pathway, that could be treated with kinase inhibitors. Through multivariate analysis of survival, they revealed that the SHM subtypes conferred a prognostic impact independently from the COO classification and IPI. Moreover, a distinct clinical outcome was observed for the SHM subtypes in the same COO subtype, and interestingly, even within unclassified DLBCL. Furthermore, they identified associations of each SHM subtype with driver mutations and oncogenic signalling pathways, proposing the possibility of choosing targeted therapy. Thus, SHM pattern represents a marker for the molecular and clinical classification of DLBCL [55]. 

Also Arthur et al. in 2018 focused on SHM activity and analysed through WGS a discovery cohort of 153 DLBCL tumour/normal pairs, and performed data validation on an internal validation cohort of 338 cases and on an external validation cohort of over 1000 additional cases to find frequently mutated coding and non-coding loci, likely targeted by aberrant SHM [56,73]. Through further analysis of matched RNA sequencing (RNA-seq) data, they suggested the potential cis-regulatory effects on coding genes of the alterations found [71,74]. These analyses revealed recurrent mutations in the 3’UTR of NFKBIZ, responsible for oncogene deregulation, and NF-κB pathway activation in the ABC subclass; instead, in the GCB subgroup they evidenced small amplifications accompanied by over-expression of FCGR2B (the Fcγ receptor protein IIB), associated with poor outcomes [56].

### 1.3. Discovering Causes of Transformation and Chemoresistence

In 2014 Pasqualucci and researchers through WES and copy-number analysis performed a pioneering work highlighting aberrations of CDKN2A/B, MYC and TP53 as major drivers of transformation of follicular lymphoma (FL) to an aggressive malignancy, typically DLBCL [58] (Table 1). Further, a subsequent work showed that one third of transformed FL harbor a MYC rearrangement [75]. More recently, Gonzalez-Rincon et al. performed targeted NGS on 22 matched samples of pre-transformed FL/transformed DLBCL patients and on 20 non-transformed FL patients [59]. Through this approach they identified several recurrently mutated genes with roles in B-cell differentiation, GC architecture and migration that were enriched at transformation, such as LRP1B, GNA13, and, in particular, POU2AF1, whose mutations seemed to characterize transformed forms rather than de novo DLBCL cases. Overall, they observed that pre-transformed FLs samples were more mutated and presented greater subclonal heterogeneity than non-transformed forms. Specifically, four genes differed between patients who did and did not show transformation: NOTCH2, DTX1, UBE2A and HIST1H1E; the mutation of these genes was related to a shorter time to transformation. With this information, the authors conclude, it could be easier to identify patients at higher risk of transformation [59].

With the purpose of investigating the pathogenic causes of chemoresistance and relapse in DLBCL, a 2014 work sequenced VDJ junctions in 14 pairs of matched diagnosis–relapse tumours [60]. The results of this study proposed two mechanisms of clonal evolution in which the early-divergent mode found two distinct clones, the diagnostic one and the relapsing one, that diverged early; and the late-divergent mode, in which relapse clones descended directly from diagnostic clones with minor divergence. Indeed, they identified in epigenetic modifiers such as KMT2D the potential early driving mutation targets, and in immune escape alterations the relapse-associated events [60]. A following research analyzed 38 R/R DLBCL biopsies obtained at the time of progression after immunochemotherapy with WES and compared the obtained mutation frequencies to an unrelated cohort of 138 diagnostic DLBCLs, with the aim of identifying relapse-associated genes. Through this approach they evidenced TP53, FOXO1, MLL3 (KMT2C), CCND3, NFKBIZ, and STAT6 as top candidate genes implicated in therapeutic resistance. Indeed, they detected mutations that may affect sensitivity to novel therapeutics, such as MYD88 and CD79B mutations in a portion of R/R ABC patients, and STAT6 mutations in one third of R/R GCB patients that were associated with activated JAK/STAT signaling, increased phospho-STAT6 protein expression, and increased expression of STAT6 target genes [61]. Another research highlighted JAK-STAT pathway involvement in the relapsed samples; the authors performed WES on 14 matched primary/relapse samples from six DLBCL patients and recorded a mild increase of mutations in relapsed samples as compared to primary tumour specimens; 264 genes possibly related to therapy resistance were identified, such as tyrosine kinases, glycoproteins, and JAK-STAT pathway genes, as well as PIM1, SOCS1, and MYC, already known to be related to a risk for treatment failure [62].

Furthermore, recently two other large-scale differential multi-omics studies were conducted on R/R DLBCL patients [63]. In the former, Fornecher et al. integrated quantitative proteomics and targeted RNA-seq data obtained from a cohort of R/R versus chemosensitive DLBCL patients and listed a set of 22 transcripts/proteins pairs, whose expression levels significantly differed between the two groups. In this list appeared genes involved in metabolism such as Hexokinase 3, in the microenvironment such as IDO1, CXCL13, in cancer cells proliferation, migration and invasion or the BCR signalling pathway such as CD79B [63]. In the latter research, Rushton et al. collected samples from 134 R/R patients enrolled in three clinical trials and performed a combination of exome sequencing and target panel sequencing of lymphoma-associated genes on circulating tumour DNA (ctDNA) extracted from plasma samples and tissue biopsies [64]. They found that R/R patients were enriched for mutations in five genes; TP53, IL4R, HVCN1, RB1, and MS4A1. Apart from TP53, already described in R/R cases [54], they focused on the others showing that IL4R mutations may trigger constitutively activation of JAK/STAT signalling and were associated to inferior OS; HVCN1 modulates the B-cell receptor (BCR) function, and truncated HVCN1 isoforms have been demonstrated to enhance BCR signaling; MS4A1 encodes CD20, the target of Rituximab, and its mutations either truncate CD20, or destabilize a common transmembrane helix. Collectively, they found that DLBCL patients with such mutations present a higher risk of treatment failure [64].

### 1.4. Double Hit or Triple Hit B-Cell Lymphomas

The debate about research into aggressive mature B-cell lymphomas with MYC, BCL2 and/or BLC6 aberrations, defined as high-grade B-cell lymphoma with double or triple hit (HGBL-DH/TH), deserves a special section as their detection constitutes a principal goal according to the last WHO classification [76]. Quite recently, two research groups tried independently to recognize HGBL-DH/TH cases using gene expression signatures. Applying a gene expression–based classifier to a cohort of 928 DLBCL patients, Sha et al. identified a molecular high-grade (MHG) subgroup comprising 83 patients (9%), 75 of which were afferent to the GCB subtype [77]. They revealed a subcategory of DH lymphomas and MYC rearrangement in one half of the total. GEP analysis identified proliferative features similar to centroblasts. PFS analysis at 36 months was 37% for the MHG subgroup after R-CHOP compared with 72% for others. Indeed, DH lymphomas not afferent to the MHG subgroup showed no evidence of a worse outcome than other GCB-like patients. Furthermore, they analysed the benefits of the addition of bortezomib to standard R-CHOP therapy; the collected data suggested a possible positive response to bortezomib [77].

In another work, Ennishi et al. studied RNA-seq data deriving from 157 GCB-DLBCL cases, including 25 HGBL-DH/TH-BCL2 cases, to elaborate a gene expression signature identifying HGBL-DH/TH-BCL2 from other GCB-DLBCLs [78]. Through this approach they elaborated a 104-gene panel with which 27% of all GCB-DLBCLs were grouped by the same expression signature, even if only one half harbored MYC and BCL2 rearrangements (HGBL-DH/TH-BCL2). They evidenced that, regardless of the HGBL-DH/TH-BCL2 status, the so-called double-hit signature-positive (DHITsig+) patients were characterized by inferior outcomes after immunochemotherapy as compared to negative patients. Indeed, they projected a new NanoString assay (DLBCL90) that should be useful in routine diagnostics to easily identify DHIT-positive cases [78]. Later, Hilton et al evaluated through WES 20 DHITsig+GCB-DLBCL cases apparently lacking MYC and/or BCL2 rearrangements and revealed six tumours with cryptic MYC or BCL2 rearrangements that were FISH negative [79]. Copy-number analysis revealed MYC and MIR17HG gains or amplifications, and focal deletions of the PVT1 promoter, both of which may contribute to dysregulation of MYC and its downstream pathways. These results support the role of the GEP signature for identifying GCB-DLBCL with poor outcomes [79].

Through the expression signatures found, most HGBL-DH/TH were identified, emphasizing that, regardless of genetic or epigenetic aberrations, these patients present similar gene expression features. This observation highlights the concept that the mechanisms of alteration of the physiologic function of MYC and/or BCL2 are diverse and still emerging. Apart from structural aberrations such as translocation or gene amplification, the MYC role can be altered by transcriptional and post-transcriptional modifications, by the activation of enhancer/superenhancer elements [80,81] as well as by mutations [82]. So, it is well known that there are diverse MYC alteration mechanisms, and other new ones are being discovered. For example, very recently Gallardo et al. showed a novel mechanism of MYC signalling aberrant activation [83]. They explored the biological consequences of overexpression of hnRNP K, an RNA-binding protein, whose expression is altered in cancer. They analysed the clinical implications of hnRNP K overexpression in 75 DLBCL patients without MYC alterations, observing hnRNP K overexpression in DLBCL patients even without MYC aberrations and its association with a short OS and PFS. Furthermore, hnRNP K overexpression in transgenic mice induced the development of lymphomas and reduced survival. Indeed, through global screening experiments and biochemical assays, they showed that hnRNP K is capable of post-transcriptionally and translationally regulating MYC. This aspect renders hnRNP K overexpressing-cells sensitive to BET-bromodomain-inhibition both in vitro and in transplantation models, opening out a new treatment strategy for DLBCL patients [83].

From a diagnostic point of view, because sometimes HGBL-DH/TH DLBCL lacks aggressive morphological and/or immunohistochemical characteristics, the doubt arises whether FISH testing for MYC, BCL2, and BCL6 genes should be performed for every DLBCL case to detect DH status. A limitation to FISH testing was proposed only for GCB and double-protein expressor (DPE) (i.e., cases with MYC and BCL2 protein overexpression) DLBCL cases, thus reducing the analyses to 15% of patients [84], but until now studies on large cohorts of patients are lacking. In their report, Scott et al. evaluated the prevalence of HGBL-DH/TH and the data resulting from FISH, COO (Lymph2Cx gene expression assay and/or Hans algorithm), and IHC testing in a large cohort of 1128 DLBCL cases deriving from three clinical trials and a population-based registry [85]. Overall, 8% of the DLBCL analysed cases were HGBL-DH/TH and mostly GCB (13.3%) rather than ABC (1.7%). They demonstrated that the MYC rearrangement (MYC-R) featured 12.2% of cases that were mostly, but not totally, GCB DLBCLs: MYC-R alone and MYC/BCL6 HGBL-DH were observed in both ABC and GCB DLBCLs; instead, MYC/BCL2 and MYC/BCL2/BCL6 HGBL-DH/TH characterized only GCB. The data collected by the authors suggested that the best method for identifying all HGBL-DH/TH tumours is to perform FISH for the MYC rearrangement for all cases; when FISH testing is positive, BCL2 and BCL6 gene aberrations should be investigated [85]. Another option is limiting FISH screening to GCB DLBCLs, thus reducing FISH experiments to half of DLBCL patients, still allowing the detection of about 99% HGBL-DH/TH with BCL2 rearrangements. However, this approach would prevent identifying rare MYC/BCL6 HGBL-DH [36,86] and ABC/non-GCB cases with isolated MYC rearrangements. Indeed, the selection of DLBCL cases based on DPE status and/or COO did not allow about 35% of all HGBL-DH to be detected. Thus, FISH screening for MYC, BCL2, and BCL6 should be performed in routine diagnostics, together with gene expression assays and NGS; alternatively, the optimum is testing for MYC rearrangements, followed by BCL2 and BCL6 analyses if the former is positive [85].

However, FISH testing for MYC rearrangements frequently does not allow breakpoint characterization and MYC-partner gene identification. In an interesting study conducted by Chong et al., targeted sequencing of MYC, BCL2, BCL6 and the immunoglobulin (IG) loci was applied in 112 DLBCL cases with a MYC aberration to explore the rearrangement at base pair resolution and to identify the partner gene identity [87]. They characterized the partner gene in 88 cases and identified a breakpoint cluster region upstream of the MYC coding region and in intron 1. In this region, mostly breaks for translocations involving IGH (80%) occurred, whereas breaks involving non genic rearrangements were located downstream of the gene locus with different partners such as IGL and IGK. They identified BCL6, ZCCHC7, and RFTN1 as recurrent MYC partners, never previously described. Indeed, they tested two commercially available FISH break-apart assays for the search of MYC rearrangements, and found discordant data in 32% of the examined patients. In HGBL-DH cases most (65%) of the MYC rearrangements presented non-IG partners and the breakpoints were located outside the genic cluster region in 72% of cases. Furthermore, patients with de novo HGBL-DH and MYC-IG aberrations featured a trend toward progression and to shorter OS as compared to patients with MYC–non-IG rearrangements, thus associating MYC rearrangement architecture to the clinical outcome [87]. More recently, another work confirmed these data: by analyzing a large cohort of 2383 DLBCL patients, Rosenwald et al. identified MYC-R in 264 (11%) cases, and evidenced that the negative prognostic impact of MYC-R is largely observed in patients with MYC DH/TH disease in which MYC is translocated to an IG partner [88].

HGBL-DH/TH lymphomas are a specific subcategory according to the WHO classification, owing to their particular worse outcome [89]. As regards DH lymphomas, MYC and BCL2 rearrangements frequently trigger the corresponding protein overexpression, characterizing a specific group called DPE lymphomas [90,91], clinically featuring rapid progression and poor outcome. Recently, a study by Uchida et al. showed the positive effect of the BCL2 inhibitor venetoclax for the therapy of this subgroup of lymphomas [92]. In vitro studies on DH and DPE lymphoma-derived cell lines revealed that the survival of neoplastic cells seems to depend on BCL2 activity rather than that of MCL1, a protein with a pro-survival function. In this context, they demonstrated that venetoclax interrupts the interaction between BCL2 and BIM, a pro-apoptotic protein, induces dephosphorylation of BCL2, and represses MCL1 protein expression. In primary lymphoma cell cultures, venetoclax was able to induce apoptosis even at low doses [92], showing venetoclax as a promising strategy for the treatment of DH-DPE lymphomas. Nonetheless, the first clinical studies showed that as monotherapy it probably has no room, and even when combined with a trail inhibitor doesn’t seem effective in relapsed DLBCL [93]. However, further investigations are needed before coming to definitive conclusions.

### 1.5. NGS Application in Clinical Practice: Liquid Biopsy

In the future liquid biopsy will likely be the tool that can render NGS investigations more feasible and realistic. Analyzing circulating cell-free DNA (cfDNA) containing DNA released by the tumour cells (ctDNA), liquid biopsy is a non-invasive investigation that, joined to NGS sensitivity and specificity, will probably revolutionize cancer diagnosis, prognosis, and treatment. ctDNA has been demonstrated to be as accurate as genotyping of the diagnostic biopsy to detect somatic mutations in DLBCL [94]; indeed, ctDNA analysis has proven able to define tumour burden [95,96] and identify prognostic and actionable biomarkers [95,96,97,98,99,100]. Indeed, it reflects the real tumour genomic heterogeneity, as demonstrated by the observation of varies mutations maybe originating from different tumour-associated localizations. Further, through liquid biopsy the response to therapy and minimal residual disease can be monitored, as well as transformation or chemoresistance emerging by tracking genetic evolution through ctDNA analysis over time [94,96]. A 2019 work conducted liquid biopsy through targeted-NGS on a set of 390 lymphoma- and cancer-relevant genes in 50 lymphoma patients in order to establish the mutation profiles of different lymphoma subtypes and evaluate the correlation between the cfDNA concentration and other clinical indexes such as serum LDH and IPI [101]. The cfDNA concentration in the plasma was significantly correlated with the clinical indices in DLBCL; indeed, the differences between GCB-DLBCL, non-GCB-DLBCL and natural killer/T-cell lymphoma were evident, confirming that NGS-based cfDNA mutation profiling is capable of discriminating different lymphoma subtypes as well as performing COO classification [96,101], thus helping to direct precision medicine actions [101].

Recently, ctDNA level measurement has recently been integrated in a new risk assessment method called CIRI (Continuous Individualized Risk Index), that dynamically evaluates individual outcome probabilities employing risk predictors obtained over time, producing real-time risk assessments during the patient’s disease course. CIRI for monitoring DLBCL patients considers a total of six risk factors, including the IPI, COO, interim imaging (iPET), along with ctDNA measurements prior to cycles one, two, and three of therapy, and has been demonstrated to improve outcome prediction compared to conventional risk models, thus enabling therapy selection in the perspective of personalized medicine [102].

### 1.6. Discovering Personalized Treatment Approaches

In the last years, the clinical management of patients with malignant lymphoma has benefited from research on tumour genomics and biology, particularly in the context of monoclonal antibodies and small molecule inhibitors [53,103,104,105,106], despite some disappointing results of phase II/III trials on some promising agents (e.g., obinutuzumab and bortezomib) [107] for which the underlying mechanisms are not well understood. Moreover, a problem of the first line studies is that in relapsed setting drugs have a short duration of responses and no plateau. Hence, any benefit seen in relapse setting does not necessarily translate in a durable increase in cure/remission in first line. Indeed, the efficacy of some drugs could be reduced because of incorrect combination with chemotherapeutic agents or insufficient dosage, as well as because of tumour-specific peculiarities [108].

In the future, NGS implementation in the routine clinical diagnostics will render tumour genetic profiling within everyone’s reach, offering the chance of a choice of tailored treatment strategies. However, despite the enormous excitement about the hypothesis of using targeted agents for patient personalized treatment, there are a series of inherent difficulties to be surmounted. Randomized trials on the addition of targeted drugs (ibrutinib, everolimus, bortezomib, and lenalidomide) to standard chemotherapy did not demonstrate a clear advantage [109,110,111,112], and in some circumstances the use of targeted agents alone has been hypothesized. In other cases, the opportunity of drug combinations has been discussed [113], as a better response can be obtained than with each drug alone. Indeed, although nowadays a number of new potent drugs are available, it is equally true that there could be a wide list of drug combinations that could be employed for patient treatment. The opportunity of using drug combinations that attack important cancer-signalling pathways at the same time from multiple fronts raises the chance of therapeutic success, especially in the treatment of tumours such as DLBCL that present with complex genetic heterogeneity. Indeed, this approach can reduce treatment resistance, which can frequently be due to pathway redundancies, cancer cell heterogeneity, and disease evolution [114,115]. A clear example that confirms this concept is R-CHOP, which has recently been demonstrated to be effective and curative thanks to low cross-resistance, rather than synergy among drugs [116].

However, today only a narrow list of approved drug combinations is available; indeed, they mostly derive from empirical clinical experience rather than rational design. To meet this need, some computational models have been developed that integrate the tumour genomic signatures with pharmacological profiles of drugs. In 2018, Preuer et al. developed a deep neural network model, DeepSynergy, to elaborate drug combinations integrating the data deriving from gene expression analysis of 39 cancer cell lines with the chemical peculiarities of 38 anti-cancer drugs [117]. However, some authors observed that this method uses large numbers of known synergistic drug combinations, frequently not providing a hypothesis of the potential mechanism of a specific drug combination synergy. Other approaches were then developed in order to identify the underlying molecular mechanisms of disease; one example is Combinatorial Drug Assembler (CDA) [118], a pathway-based model elaborated to discover drug combinations targeting pathways that overlap with tumour-enriched signalling pathways using differentially expressed genes. Another model is TIMMA [119], which identifies drugs targeting multiple driver pathways by elaborating and combining drug screening data and drug target interactions into a target inhibition network framework. However, the survival pathways to be targeted were identified based on empirical selection, not considering genomic data.

Recently, some investigators proposed a new computational system biology tool that they called DrugComboExplorer, which combines specific genomic characteristics of cancer types (i.e., signalling pathways, interactome and pharmacological data) with pharmacogenomic profiles of 5585 drugs and bioactive compounds from the NIH LINCS program (Library of Integrated Network-based Cellular Signatures) [120]. Indeed, by adopting a data-driven strategy and by combining multi-omics data (DNA seq, gene copy number, DNA methylation, and RNA-seq data) of individual cancer patients, this tool unveils new regulatory signalling pathway mechanisms (i.e., driver signalling networks) and is able to perform large-scale drug combination prediction (15,593,320 available drug combinations). In vitro validation experiments on DLBCL and prostate cancer cell lines, in order to evaluate the reliability and the predictive power of their bioinformatics tool, confirmed its utility in identifying targetable cancer driver pathways and prioritizing potential drug combinations useful to attack them [120]. 

**Figure 1:**
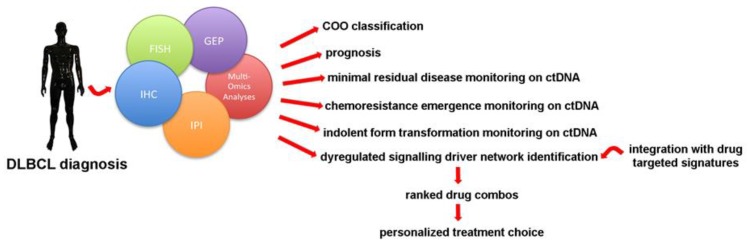
Today a series of technologies are available for DLBCL profiling. Through their integration each patient can benefit from a better diagnostic and prognostic framework, including non-invasive disease tracking on ctDNA analysis with liquid biopsy. From the perspective of personalized medicine, the treatment option will be stitched onto the patient after a multi-omic analysis of the disease’s specific biologic features. This information will be then combined with drug-specific peculiarities to generate a list of targeted drug combinations for the choice of the best therapy for each patient.

Surely, this kind of approach is still in its infancy as the authors admit, but further investigations are ongoing to try to apply these models to specific cancer cases in clinical practice in order to identify personalized drug combinations and more efficient treatment plans for individual patients [120]. Then, large scale collaborations should be scheduled integrating mulit-omics data, Bayesian trial design, and early shared endpoints based on, for example, CIRI or any interim guided models to test in vivo the reliability of these drug combination-predicting models (Figure 1).

## 2. Conclusions

The attentive observer has surely realized that there is currently a dichotomy between the potentialities deriving from the recent discoveries for DLBCL diagnosis, prognosis, and treatment, and patient management in real clinical life. Despite discoveries of new drugs, R-CHOP remains the standard treatment approach, and targeted therapy is considered mostly for R/R patients. This circumstance will probably persist for a while, but when the biological background becomes clearer, when genome-wide screening has become within everyone’s reach, and when targeted drugs have demonstrated their real benefits, personalized medicine will become feasible also for DLBCL. 

In this panorama, a great improvement in clinical management of patients will certainly derive from the synergy of data obtained from liquid biopsy, providing information about therapy options stitched onto the patient’s specific disease. Probably, today we are still far from this goal, as standardizations and clinical trial designs are still needed to render molecularly driven approaches really achievable. In any case, the bases are there, allowing us to pursue the goal of realizing targeted therapy for DLBCL.

## Figures and Tables

**Table 1:** Summary of the most relevant recent studies about DLBCL molecular classification and prognostication.

Reference	Kind of Study	Main Molecular Findings	Clinical Implications
New Prognostic Biomarkers and Models			
Reddy et al., *Cell* 2017 [49]	Exome and transcriptome sequencing of 1001 DLBCL cases	Identification of 150 driver genes set, definition of a prognostic model better than current ones	- MYC mutations or aberrant expression: worst prognosis - CD70 alterations: better outcome
Schmitz et al., *NEJM* 2018 [50]	WES, RNA-seq, gene copy number analysis and targeted sequencing of 372 genes in 574 DLBCL cases	Development of a specific algorithm identifying four genetic subtypes: - MCD (MYD88L265P and CD79B mutations), - BN2 (BCL6 fusions and NOTCH2 mutations), - N1 (NOTCH1 mutations), - EZB (EZH2 mutations and BCL2 translocations)	- BN2 and EZB: favourable outcome - MCD and N1: inferior outcome - MCD and BN2 subtypes depend on BCR signalling pathway activation (targeted therapeutic option)
Chapuy et al., *Nat Med* 2018 [51]	WES and targeted sequencing on 304 DLBCL patients	Identification of five DLBCL subsets: - C1 (NOTCH2 mutations) - C2 (TP53 and CDKN2A alterations, genomic instability) - C3 (PTEN, KMT2D, CREBBP, and EZH2 aberrations) - C4 (BCR–PI3K, NF-κB, or RAS–JAK pathway alterations, BRAF, STAT3, CD83, CD70, and CD58 mutations) - C5 (BCL2, MYD88L265P, CD79B, PIM1, and PRDM1 alterations)	- C1: low-risk - C2: poor outcome - C3: poor outcome - C4: favourable outcome - C5: unfavourable outcome
Vermaat et al., *Haematologica* 2019 [52]	NGS, allele-specific PCR and FISH on 250 DLBCL cases	Identification of: - MYD88 and CD79B mutations in 29.6% and 12.3% - MYC, BCL2, and BCL6 rearrangements in 10.6%, 13.6%, and 20.3%, respectively	MYD88 mutations: adverse prognostic impact
Jain et al., *Sci. Transl. Med* 2019 [53]	DNA copy number analysis of 1000 DLBCL cases	Identification of 18q21.2 gains as the most frequent genetic alteration in the ABC-like group, with involvement of TCF4 (E2-2) transcription factor gene	The inhibition of TCF4 activity through BET inhibitors could be employed in the treatment of this patient subset
Intlekofer et al., *Blood Cancer* 2018 [54]	Targeted NGS on 198 DLBCL cases	Identification of a median number of six genetic aberrations per case, with 97% of patients presenting at least one alteration and 54% of cases more than one (e.g., MYD88, CREBBP, CD79B, EZH2)	- Less common aberrations (BRAF, CD274 (PD-L1), IDH2, and JAK1/2) could be employed as potential therapeutic targets - TP53 alterations: more frequently associated to lack of response to first-line chemotherapy and involved in R/R DLBCL
Alkodsi et al., *Leukemia* 2019 [55]	WGS, RNA-seq, and gene expression from literature DLBCL cohorts	The expression of 36 SHM target genes identifies four SHM subtypes: - SHM1 (BCL2, MYC, and chromatin modifying genes aberrations) - SHM2 (BCR signalling pathway mutations) - SHM3 (JAK-STAT pathway mutations) - SHM4 (BCL6 fusions and mutations in CD70 and BCL10)	- SHM1: poor outcome after standard R-CHOP therapy - SHM2: worst outcome, could be treated with kinase inhibitors - SHM3: better outcome to standard cure - SHM4: worst outcome, similar to SHM2
Arthur et al., *Nat. Commun.* 2018 [56]	Integrative analysis of whole genomes, exomes, and transcriptomes on thousands of DLBCL cases	Identification of: - recurrent NFKBIZ 3’ UTR mutations causing NF-κB pathway activation in the ABC subgroup - Small amplifications associated with over-expression of FCGR2B, in the GCB subgroup	These results revealed new driver DLBCL mutations, improving diagnostic assays and offering new possibilities for the development of targeted therapeutics
Wang et al., *Carcinogenesis* 2019 [57]	WES on 22 early stage DLBCL and validation on 35 primary DLBCL cases	Identification of two MATH score classes: low and high MATH score groups according to the median expression level	- The higher MATH score group was associated with a higher risk of progression - The MATH score has a prognostic value that could be considered in the management of DLBCL patients
Causes of Transformation and Chemoresistance			
Pasqualucci et al., *Cell Rep.* 2014 [58]	WES and SNP array analysis on 12 FL samples at diagnosis and on 39 transformed FL	Identification of CDKN2A/B, MYC and TP53 as major drivers of transformation of FL to an aggressive malignancy, typically DLBCL	The genomic profile of transformed FL shares similarities with de novo DLBCL-GCB but also displays unique gene mutations with diagnostic and therapeutic implications
González-Rincón et al., *PLoS One* 2019 [59]	Targeted NGS on 22 pre-transformed /transformed and on 20 non-transformed FL cases	Transformed FL are characterized by several recurrently mutated genes with roles in B-cell differentiation, GC architecture and migration (LRP1B, GNA13 and POU2AF1)	- Four genes differed between patients who did and did not show transformation (NOTCH2, DTX1, UBE2A and HIST1H1E) - the mutation of these genes was related to a higher risk of transformation
Jiang et al., *Genome Biol.* 2014 [60]	High-throughput sequencing of rearranged VDJ junctions in 14 pairs of matched diagnosis-relapse DLBCL	Two proposed mechanisms of clonal evolution: - the early-divergent mode with two distinct clones (the diagnostic and the relapsing one) that early diverged; - the late-divergent mode, in which relapse clones descended directly from diagnostic clones with minor divergence	Although DLBCL relapse may result from multiple tumour evolutionary mechanisms, each mechanism could provide rationale for therapies
Morin et al., *Clin. Cancer Res.* 2016 [61]	WES on 38 R/R DLBCL biopsies and on an unrelated cohort of 138 diagnostic DLBCLs	Identification of TP53, FOXO1, MLL3 (KMT2C), CCND3, NFKBIZ, and STAT6 as top candidate genes implicated in therapeutic resistance	Detection of mutations (MYD88 and CD79B) that may affect sensitivity to novel therapeutics
Nijland et al., *Cancers (Basel)*. 2018 [62]	WES on 14 matched primary/relapse samples from six DLBCL patients	Identification of 264 genes possibly related to therapy resistance, including tyrosine kinases, transmembrane glycoproteins, and genes involved in the JAK-STAT pathway	Identification of resistance-related genes such as PIM1, SOCS1, and MYC, that confer a risk for treatment failure
Fornecker et al., *Sci. Rep.* 2019 [63]	Integrated quantitative proteomics and targeted RNA-sequencing in 8 R/R DLBCL cases versus 12 chemosensitive DLBCL patients	Identification of a set of 22 transcripts/proteins pairs, whose expression levels significantly differed between the two analysed groups	Identification of new biomarkers related to chemoresistance, new potential drug targets: Hexokinase 3, IDO1, CXCL13, S100 proteins, CD79B
Rushton et al., *Hematol. Oncol.* 2019 [64]	WES and targeted NGS on plasma samples and tissue biopsies from 134 R/R patients	R/R patients were enriched for mutations in five genes: TP53, IL4R, HVCN1, RB1 and MS4A1	DLBCL patients with mutations in these five genes present a higher risk of treatment failure

Abbreviations: WES, whole-exome sequencing; RNA-seq, transcriptome sequencing; NGS, next-generation sequencing; R/R, relapse or refractory; MATH, mutant-allele tumour heterogeneity; SNP, single nucleotide polymorphism; FL, follicular lymphoma; VDJ, Variable Diversity Joining.
